# Using NIATx strategies to implement integrated services in routine care: a study protocol

**DOI:** 10.1186/s12913-018-3241-4

**Published:** 2018-06-08

**Authors:** James H. Ford, Eric L Osborne, Mehret T. Assefa, Amy M McIlvaine, Ahney M King, Kevin Campbell, Mark P McGovern

**Affiliations:** 1 School of Pharmacy – Social and Administrative Sciences Division, University of Wisconsin – Madison, 777 University Ave, Madison, WI 53705 USA; 2 Office of Behavioral Health and Managed Care, Division of Behavioral Health and Recovery, Washington State Department of Social and Health Services, Olympia, WA 98504 USA; 3 Center for Behavioral Health Services and Implementation Research, Division of Public Health & Population Sciences, Department of Psychiatry & Behavioral Sciences, Stanford University School of Medicine, 1520 Page Mill Road, Palo Alto, CA 94304 USA; 4 Office of Behavioral Health and Prevention, Division of Behavioral Health and Recovery, Washington State Department of Social and Health Services, Olympia, WA 98504 USA; 5 Washington State Health Care Authority, Olympia, WA 98501 USA; 6 Division of Public Mental Health & Population Sciences, Department of Psychiatry & Behavioral Sciences, Division of Primary Care and Population Health, Department of Medicine, Stanford University School of Medicine, 1520 Page Mill Road MC5265, Palo Alto, CA 94304 USA

**Keywords:** Co-occurring disorders, Integrated treatment, NIATx implementation strategies, DDCAT

## Abstract

**Background:**

Access to integrated services for individuals with co-occurring substance use and mental health disorders is a long-standing public health issue. Receiving integrated treatment services are both more effective and preferred by patients and families versus parallel or fragmented care. National policy statements and expert consensus guidelines underscore the benefits of integrated treatment. Despite decades of awareness, adequate treatment for individuals with co-occurring substance use and mental health disorders occurs infrequently. The underlying disease burden associated with alcohol, illicit and prescription drug problems, as well as mental health disorders, such as depression, posttraumatic stress disorder and schizophrenia, is substantial.

**Methods:**

This cluster randomized controlled trial (RCT) is designed to determine if the multi-component Network for the Improvement of Addiction Treatment (NIATx) strategies are effective in implementing integrated services for persons with co-occurring substance use and mental health disorders. In this study, 50 behavioral health programs in Washington State will be recruited and then randomized into one of two intervention arms: 1) NIATx implementation strategies, including coaching and learning sessions over a 12-month intervention period to implement changes targeting integrated treatment services; or 2) wait-list control. Primary outcome measures include: 1) fidelity - a standardized organizational assessment of integrated services (Dual Diagnosis in Addiction Treatment [DDCAT] Index); and 2) penetration - proportion of patients screened and diagnosed with co-occurring disorders, proportion of eligible patients receiving substance use and mental health services, and psychotropic or substance use disorder medications. Barriers and facilitators, as determinants of implementation outcomes, will be assessed using the Consolidated Framework for Implementation Research (CFIR) Index. Fidelity to and participation in NIATx strategies will be assessed utilizing the NIATx Fidelity Scale and Stages of Implementation Completion (SIC).

**Discussion:**

This study addresses an issue of substantial public health significance: the gap in access to an evidence-based practice for integrated treatment for individuals with co-occurring mental health and substance use disorders. The study utilizes rigorous and reproducible quantitative approaches to measuring implementation determinants and strategies, and may address a longstanding gap in the quality of care for persons with co-occurring disorders.

**Trial registration:**

ClinicalTrials.gov NCT03007940. Registered 02 January 2017 – Retrospectively Registered

**Electronic supplementary material:**

The online version of this article (10.1186/s12913-018-3241-4) contains supplementary material, which is available to authorized users.

## Background

Access to integrated treatment services for individuals with co-occurring substance use and mental health disorders is a longstanding problem in behavioral health care [1, 2]. The provision of integrated mental health and substance use services during the same treatment episode by the same clinical provider addresses national policy statements and expert consensus guidelines underscoring the benefits of integrated treatment [1–3]. It is also preferred by patients and families [4]. However, a significant gap remains between the availability of “one-stop” integrated services and the actual receipt of integrated services for individuals with co-occurring disorders. Despite increased awareness, adequate integrated treatment for individuals with co-occurring substance use and mental health disorders occurs infrequently [5, 6]. In the United States, only 18% of specialty addiction programs and 9% of mental health programs offer integrated services [7]. Availability of integrated services is not associated with receipt of services. Consumers with co-occurring disorders report only receiving integrated services between 7 to 9% of the time [8, 9]. However, it is unclear if these individuals had their co-occurring disorders addressed in treatment at the same time or if they even received integrated services.

The current system represents an undesired but chronic, systemic artifact for policymakers and treatment providers, and even more so for families and individuals suffering from co-occurring disorders [6, 10–12]. The resulting fragmented system of care requires multiple provider interactions and integrated care is almost non-existent. The disease burden associated with co-occurring disorders represents a substantial public health concern [13–20]. Inadequate access to effective integrated treatment results in poorer public health and societal outcomes [21–25]. The impact on the United States healthcare system is significant. By 2020, annual expenditures in the US for co-occurring substance use and mental health disorders are projected to reach $281 billion [26].

Despite these facts, integrated services for individuals with co-occurring disorders are not being widely implemented in behavioral health organizations [27, 28]. Research on effective implementation of evidence-based approaches to integrated treatment for co-occurring disorders is sorely needed [29–31]. This research study addresses this gap utilizing an implementation science approach.

## Conceptual model and theoretical justification

**Fig. 1:**
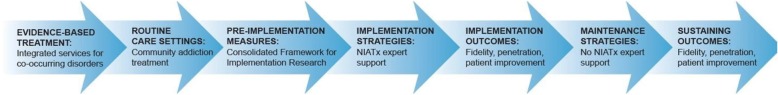
Unified Conceptual Model The model outlines the integration and use of objective measures across three frameworks: determinant (Consolidated Framework for Implementation Research); evaluative (Proctor’s implementation outcome taxonomy); and process (Stages of Implementation Completion [SIC]) with NIATx Implementation Strategies to implement integrated services for co-occucring disorders in community addiction treatment programs

Implementation science holds the methodological key to the effective implementation of evidence-based approaches to integrated treatment for co-occurring disorders. This research utilizes objective measures across three types of frameworks (determinant, evaluative and process) outlined by Per Nilsen [32] to create a conceptual unified implementation research model (Fig. 1).

The Consolidated Framework for Implementation Research (CFIR), an evaluative framework, articulates factors impacting the success or failure of an implementation strategy [33–39]. In this study, we will focus on four CFIR dimensions (Outer Setting, Inner Setting, Characteristics of the Intervention and the Individual) that are particularly salient during pre-implementation of organizational change. However, the absence of a quantitative measure is a limitation of the CFIR [40, 41]. Therefore, for this study, we will use the quantitative instrument we developed of the CFIR items across these four dimensions to assess the presence of potential facilitators or barriers to the implementation process, the CFIR Index.

Proctors’ implementation taxonomy represents an evaluative framework that differentiates between implementation, service and patient outcomes [42]. This study will focus on implementation (fidelity and penetration) and patient care outcomes. The final component will examine implementation strategy participation which has suffered from a lack of clarity in definition, description, documentation and terminology precision [43–47]. The study will use the Stages of Implementation Completion (SIC) as a process framework to assesses implementation strategy participation by tracking a list of milestone activities and measuring the proportion of completed activities and the duration (time) to completion [48–50]. This study will adapt the SIC to assess program completion of the NIATx implementation strategy.

## NIATx implementation strategy

The NIATx implementation strategies will be incorporated into this conceptual model (see Step 4 in Fig. 1) to determine the effectiveness of NIATx in implementing integrated services for persons with co-occurring substance use and mental health disorders. NIATx combines process improvement tools and techniques (e.g., consumer-centered walk-through and PDSA rapid change cycles) with quality improvement interventions such as coaching, learning sessions, and “interest circle” calls [51–53]. NIATx implementation strategies have been widely adopted and successfully utilized to improve and sustain access to care and addiction medications [54–59]. The NIATx200 study dismantled three components of the NIATx implementation strategy (learning session, coaching, and interest circle calls) or a combination of all three to determine key elements for improving wait time, admissions and retention [60]. This dismantling of NIATx found important differences by implementation strategy and outcome; however, it was in fact an “intent-to-treat” analysis [61]. Fidelity and extent of variation within each component were not assessed in the NIATx200 study.

Two studies provide evidence for NIATx as an effective implementation strategy to improve access to integrated treatment for individuals with co-occurring disorders. The use of unspecified “NIATx-like” implementation strategies (e.g., PDSA cycles, change champion and team, coach, and process/outcomes measurement) in 54 treatment agencies in five states significantly predicted changes in DDCAT Total Score [62]. In an “open-label” single group repeated measures design, eight community addiction treatment agencies participated and received expert NIATx support during a six-month timeframe. Measures included pre and post DDCAT assessments and changes in Addiction Severity Index (ASI) substance use and psychiatric severity scores. Results indicated that seven of the eight agencies made significant improvements in integrated service capacity over six months (change in DDCAT Total Score range 0.5 to 0.8), and patient level data (range in n by program: 19 to 588) revealed corresponding positive changes in ASI drug, alcohol and psychiatric severity composite scores [63]. These studies provided a compelling signal for more rigorous and controlled research, which is absolutely necessary to advance, with scientific confidence, the use of NIATx to integrate services. This research study will address that gap by determining if NIATx strategies are effective in implementing integrated services for persons with co-occurring substance use and mental health disorders.

## Methods/study design

### Overview

**Fig. 2:**
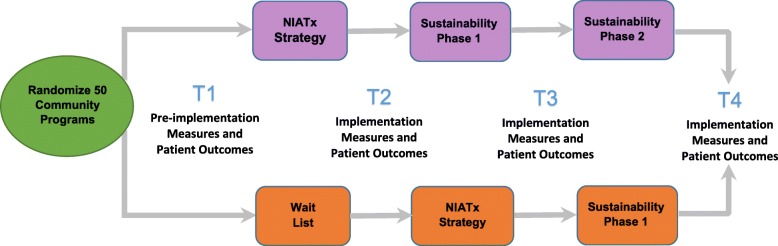
NIATx Implementation Strategy Study Design. The community programs are randomized to NIATx (Cohort 1) or Wait List (Cohort 2) with four data collection time points

The project represents a collaboration between Stanford University, University of Wisconsin-Madison, and the Division of Behavioral Health and Recovery (DBHR) located in the Washington State Department of Social and Health Services. The study uses a cluster randomized wait-list control group design. Fifty community-based addiction treatment programs located in the State of Washington will be assigned to one of two cohorts (Fig. 2) during an index 12-month period: 1) NIATx implementation strategies (Cohort 1), or 2) wait-list control (Cohort 2).

**Fig. 3:**
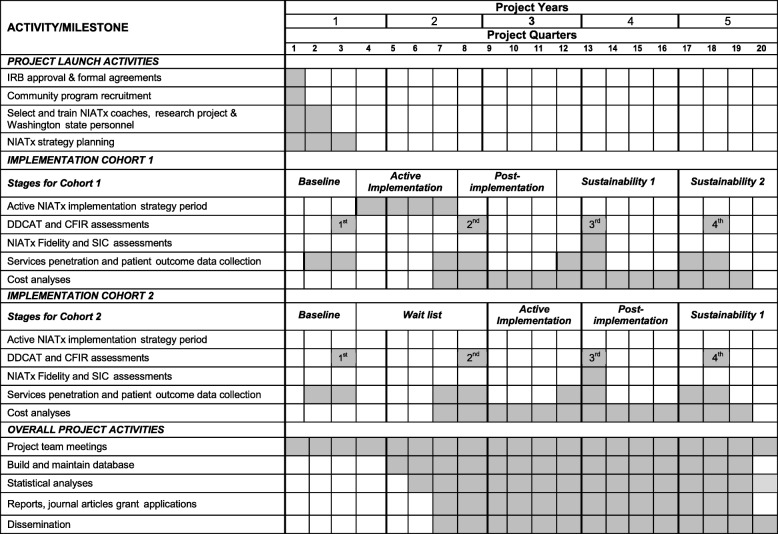
NIATx Implementation Study Project Timeline. The study project timeline is organized by activities associated with a) project launch, b) cohort 1, c) cohort 2, and d) overall project activites over the five year study period

The programs will use the NIATx implementation strategy to implement changes targeting integrated treatment services. The study will assess the effectiveness of the NIATx implementation strategies to improve integrated services for persons with co-occurring substance use and mental health disorders. Hypothesized effects are that relative to the wait-list, NIATx strategies will improve implementation (penetration and fidelity) and patient care outcomes (Aim 1 and Aim 2). Variation in the extent of and fidelity to the NIATx implementation strategies will be examined across the entire sample (Aim 3). The specific aims and hypothesis are detailed in Table 1. Figure 3 shows the overall study timeline. Recruitment began in April 2016 and the active intervention period for Cohort 2 ends in June 2018.

**Table 1:** Study Specific Aims and Hypotheses

Aim	Hypotheses
Specific Aim 1: Relative to wait-list, to determine if NIATx strategies improve *implementation fidelity outcomes*.	H1: *NIATx strategies will produce increased integrated service fidelity at the program level*.
Specific Aim 2: Relative to wait-list, to determine if NIATx strategies improve *implementation penetration outcomes*.	H2: *NIATx strategies will produce increased penetration rates in integrated services, evidenced by proportion of program patients screened, diagnosed and receiving integrated medication and psychosocial services.*
Specific Aim 3: Across entire sample, to evaluate variation in the *extent of and fidelity to NIATx* strategies.	H3: *Programs with more facilitating factors, articulated by the Consolidated Framework for Implementation Research (CFIR) Index dimensions will be more likely to complete the requisite tasks of the NIATx protocol and do so with greater fidelity.*

### NIATx implementation strategy

Programs in the NIATx intervention will be assigned a NIATx trained process improvement coach who leads the active implementation phase. Over a 12-month active implementation period, the coach works with executive directors, change leaders and teams. Coaching includes a one-day site visit and individual monthly phone conferences (10 h total) with each program.

Prior to the site visit, the coach will introduce the project, review initial DDCAT results, discuss how to conduct a walk-through [64] and set the stage for the site visit. The site visit will use a standardized agenda to ensure fidelity. During the visit, the coach will meet with executive leadership, review the walk-through and DDCAT assessment results, and train staff on the use of the NIATx implementation strategies. With their coach, the program will utilize results from the DDCAT assessment to identify areas for improvement, implement change projects and assess their impact.

After the site visit, the coach will conduct bi-monthly coaching calls with their assigned programs for the 1st quarter of the implementation period and monthly calls thereafter. In the individual calls, the coach and change team will review change projects, discuss successes, and identify new change projects. In addition to individual coaching calls, the coach provides support through learning sessions and group coaching calls.

Two group calls, moderated by the coach, will involve change leaders from multiple programs and provide an opportunity for peer-to-peer sharing. On these calls, the change leaders will discuss common change-related issues, progress, and exchange innovative implementation strategies with their peers. The calls will also allow the coach to share new strategies and discuss implementation issues such as sustainment of organizational change.

The study will include two one-day coach-led learning sessions for all programs within a cohort. Learning sessions promote peer-to-peer sharing about specific goals and objectives using a tailored agenda. The first learning session will teach programs how to use NIATx process improvement strategies through skill development activities such as how to identify change opportunities, develop PDSA cycles and effective use of data to drive change. The second learning session will include program presentations about change efforts and discuss how to develop sustainment plans to continue to improve integrated services.

Coach supports will ensure that NIATx is delivered with fidelity to all participating programs. Supports include: a one-day coach training session at the study start to review objectives, provide NIATx refresher and review how to interpret DDCAT results in order to design change projects; a standardized site visit agenda; and a standardized coach report to capture program interactions. In addition, the coaches will participate in monthly calls with the PI (Dr. Ford) to review progress, discuss issues, receive advice from peers, share promising practices and clarify any research issues.

### Eligibility and recruitment

Programs will be recruited from the population of 486 licensed addiction treatment programs in Washington State. Eligibility criteria included: offering outpatient and/or intensive outpatient services; tax-exempt, government status or at least 50% publically funded; and no prior participation in NIATx research studies. Public mental health and private addiction treatment programs were excluded because they are not required to utilize the state clinical information system, and therefore cannot provide the necessary standardized project data. A study recruitment letter will be created and distributed by Division of Behavioral Health and Recovery staff to all eligible programs.

### Randomization

The randomization sequence will be generated by the study biostatistician and concealed from the researchers conducting study assessments. An equal number of programs will be randomized to each study arm. However, the coaches will not be blinded to the results of randomization as they will be assigned to programs in Cohort 1 after the baseline DDCAT assessment is completed. After the baseline DDCAT assessment is completed, the program along with their coach, will be notified of their intervention assignment (Cohort 1) or the program will be notified that they have been assigned to the wait-list control group (Cohort 2).

### Data collection/variables

The proposed research will explore the impact of the NIATx implementation strategies on positive changes in an implementation fidelity outcome (Aim 1) assessed by the Dual Diagnosis Capability in Addiction Treatment (DDCAT) Index. The DDCAT (Version 4.0) is a 35-item observational benchmark measure of program level dual diagnosis capability. Items are rated on a 5-point scale on degree of integration to generate a total score and scores on seven dimensions [62, 65, 66]. Two studies provide evidence that improvements in or higher DDCAT scores impact patient outcomes. In a study of 185 substance abuse providers, individuals receiving treatment in clinics with higher DDCAT scores had significantly longer length of stay and although not significant, attend four additional treatment sessions [66]. Results from an “open-label” single group repeated measures design (*n* = 8 community addiction treatment agencies) found that overall DDCAT scores increased on average 0.56 points which was associated with corresponding changes in standardized Addiction Severity Index composite severity scores in the psychiatric (μ = 0.034 ± 0.075), alcohol (μ = 0.007 ± 0.120) and drug (μ = 0.014 ± 0.091) problem categories [63]. The DDCAT will be assessed for all participating programs at four distinct time points (Table 2).

**Table 2:** Implementation and Fidelity Measures and Frequency of Data Collection

			Data Collection Time Periods			
Aim	Construct	Measure	Baseline	Post-Implementation	Sustainment Period 1	Sustainment Period 2
1	Integrated Services: Fidelity	Dual Diagnosis Capability in Addiction Treatment (DDCAT) Index^1^	X	X	X	X
2	Integrated services: Patients Screened	Proportion of program patients: screened using Gain Short Screener (GSS)	X	X	X	X
2	Integrated Services: Medications	Number of patients receiving a psychotropic or substance use disorder medication	X	X	X	X
2	Integrated Services: Chemical Dependency Services	Number of patients receiving chemical dependency services	X	X	X	X
2	Integrated Services: Mental Health Services	Number of patients receiving mental health services	X	X	X	X
3	Program Facilitators and Barriers to Implementation	Consolidated Framework for Implementation Research (CFIR) Index^1^	X	X	X	X
3	NIATx Stages of Implementation Completion^2^	NIATx strategy fidelity and extent of and duration to complete activities (SIC)		X		
3	NIATx Fidelity Scale^3^	NIATx strategy fidelity and extent of and duration to complete activities (SIC)		X		
1	Program characteristics^1^	Program Size (Admissions), Program Type, ASAM Levels of Care, Payment Sources	X	X	X	X
2	Patient Characteristics	Age, Gender, Race, Ethnicity	X	X	X	X

State of Washington staff will be trained on how to conduct DDCAT and CFIR Index assessments by co-PI (McGovern). Two-person teams will schedule and conduct each DDCAT assessment. Additional training or consultation will help answer questions identified at the site visits. Program characteristics are collected during the DDCAT assessments

Data will be collected by the state of Washington staff and NIATx coaches using standardized instruments for each program participating in the study

NIATx Fidelity scoring (Total and 7 subscale scores are organized by preparation, implementation and sustainment phases) will be assessed by two person teams at the end of the active implementation period for each program. Data sources will include a composite of interviews, review of walk-through results, change project forms, coach notes and sustainability plans

At the time of the study, Washington State was transitioning to Managed Care Organizations (MCOs) to pay for the delivery of substance use disorder (SUD) and mental health (MH) services. The transition involved integrating data from two systems: 1) the Treatment and Assessment Reports Generation Tool (TARGET), covering SUD clients and services; and 2) the Mental Health Consumer Information System (MH-CIS), covering community MH clients and services into a new Behavioral Health Data System (BHDS). The study will capitalize and leverage the State of Washington’s experience in utilizing standardized state-wide clinical management information databases for addiction and mental health treatment [67–70]. Implementation penetration outcomes will assess changes in the proportion of patients screened, diagnosed and receiving integrated psychosocial or medication services (Aim 2). The services data will be extracted from the BHDS as well as TARGET and MH-CIS legacy systems. It will include de-identified client level data for all patient admissions to study programs within a 45-day window before and after each DDCAT assessment date (Additional file 1). The information will be transferred to the study team using appropriate security protocols.

The CFIR Index operationalized four CFIR dimensions (Characteristics of the Intervention; Outer Setting, Inner Setting, Characteristics of Individuals) to create an objective rating scale to evaluate pre-implementation factors as moderators of the implementation process over time, and as factors in sustainability (Aim 3). The index has good preliminary psychometric properties [71, 72]. Summary ratings from the CFIR Index dimensions may predict fidelity to and extent of completion of the NIATx strategies (Aim 3). Data collection for the CFIR Index will follow the same schedule as the DDCAT assessment.

Fidelity to and participation in the NIATx implementation strategies will be assessed using two exploratory scales specifically developed for this study: NIATx Fidelity Scale, and the NIATx Stages of Implementation Completion (NIATx SIC). The NIATx Fidelity Scale includes 19-items designed to assess adherence to the NIATx model on a 5-point scale from 1- No evidence to 5-Extensive evidence. The NIATx SIC is based on a modified version of the SIC and is organized into three phases: Pre-implementation, Implementation and Sustainment (Table 3). Program driven activities will be scored and count toward both duration (number of days) and proportion (number of scored activities completed/total number of scored activities possible) within a given phase of the NIATx SIC. The use of these scales will be utilized to assess variation in the extent and fidelity to which NIATx strategies are delivered.

**Table 3:** Overview of the NIATx Stages of Implementation Checklist

NIATx SIC Phases and Stages in Each Phase	# of Items	Examples of NIATx SIC Elements
Program Characteristics	17	Program Size, Type, Primary Focus
Pre-Implementation Phase Stage 1: Engagement	6	Invite Date, Contacts before Accept
Pre-Implementation Phase Stage 2: Consideration of Feasibility	8	DDCAT Assessment Date, Contacts
Pre-Implementation Phase Stage 3: Readiness Planning	18	Initial Coach Engagement & NIATx Webinar, Change Leader Appointed
Implementation Phase Stage 4: Staff Hired and Intro Training	5	Change Team Identified, Coach Site Visit
Implementation Phase Stage 5: Fidelity Monitoring & Tracking in Place	4	Review of Walkthrough, Project Selection
Implementation Phase Stage 6: Services & Consultation to Services Begin	4	Collect baseline data, Start Change Project
Implementation Phase Stage 7: Model Fidelity & Staff Competence & Adherence Tracked	Varies by Program	Change Projects, Change Cycles per Project, Coaching Calls, Peer to Peer Meeting Attendance
Sustainability Phase Stage 8: Competency	10	Continue use of NIATx Implementation Strategies, NIATx Fidelity Score

A program self-reported operational survey will collect information about average staff hourly salaries (baseline and follow-up), and staff time and cost spent on NIATx implementation as well as information about the impact on operational revenues and costs (follow-up). The survey information will be utilized in the economic analysis evaluating implementation activity and resource costs.

### Data and power analysis

The analyses will include a quantitative assessment of Aims 1 to 3 and an economic cost analysis. Table 1 outlines the study aims and hypotheses. The data and power analysis approaches are presented in sequence for each aim by hypothesis for the experimental comparison only, as the pooled-group analyses have inferential limitations. Power was assessed using SamplePower 3.0 [73].

#### Specific aim 1

For hypothesis 1, the program is the unit of analysis, and DDCAT (fidelity) scores represent the dependent variable and the cohort assignment (NIATx vs. wait-list) is the independent variable. A two tailed analysis of covariance (α = 0.05) with 25 programs per cohort group will compare post- implementation mean fidelity scores between groups, with pre-implementation scores as the covariate. With a correlation between pre- and post- implementation DDCAT scores equal to 0.5, this analysis has power (β = .86) to detect a large effect. Prior research concerning one-year changes in DDCAT scores suggests that we can expect a large effect [62].

#### Specific aim 2

The date of the DDCAT assessment will serve as the index date for hypothesis 2 (Aim 2). All patients admitted to each program, 45 days before and 45 days after the DDCAT assessment date, will be extracted from the state administrative databases. The outcomes will be the proportion of program patients: 1) screened, 2) diagnosed, and 3) receiving integrated medication and psychosocial services, compared to ad hoc strategies (wait-list comparison sites). Each outcome has a value of 1 (yes) or 0 (no), and interest is in the difference in the rate of each outcome, accounting for the clustering of observations within sites. This analysis calls for a multi-level logistic regression model. The observations at each time point are independent, and therefore this is not a longitudinal (repeated measures) analysis. Instead, there are four independent groups (NIATx pre-implementation, NIATx post-implementation, wait-list pre-implementation, and wait-list post-implementation) in a 2 (group) by 2 (time) analysis, with primary interest in the group by time interaction. Due to the large number of observations, a logistic regression has power equal to 0.75 to detect a small effect (OR = 1.5; .10 difference in proportions) and power of 1.0 to detect a medium effect (OR = 2.33; .20 difference in proportions). These power estimates are based on standard logistic regression. We will apply a correction to the standard errors to adjust for the interclass correlation at the site level in order to avoid Type I errors due to the dependence of the clustered observations [74].

#### Specific aim 3

Aim 3 will evaluate variation in the extent of, and fidelity to, NIATx strategies. The specific hypothesis is that programs with more facilitating factors, evaluated using the CFIR Index dimensions, will be more likely to complete the NIATx protocol and to do so with greater participation and fidelity. The programs are the unit of analysis, and the primary predictor variable is the number of factors that support implementation. There are two dependent variables, a continuous variable indicating the proportion (%) of the 22 NIATx tasks completed (SIC) and a continuous variable indicating the degree of fidelity to the NIATx protocol (1 to 5-point scale). Multiple regression analyses will be used to evaluate both outcome variables. Characteristics of the sites that are associated with the outcomes will be added as covariates in the regression models in order to evaluate the effect of the CFIR Index dimensions (e.g., Perceptions of the System and Community Score) after controlling for other predictors. Power for the two-tailed multiple regression analysis (α = 0.05) across 50 programs, and 5% of the variance explained by the covariates, is 79% power to detect a change in R^2^ of 15 and 82% power to detect a change in R^2^ of 16%, when adding the primary predictor to the model.

##### Cost analysis

The economic cost analysis consists of two components. First, we will measure costs required to support participation in the implementation strategies (active and wait-list). In the second component, the cost analysis will examine potential changes in program finances (revenue and expenses) associated with delivering more integrated services. In contrast to the economic costs of integrating services, which includes costs not faced directly by the program are not part of any program’s operating budget, and thus savings to outside entities do not make interventions more feasible unless these savings are shared by the program. The cleanest and most comprehensive measure of the costs to a program providing integrated treatment is the pre-post (NIATx) change in total costs netting out any increased revenue, or adding in any loss in revenue, for the program. We will duplicate our successful earlier collection of costs, revenue, and admission information from each program [75]. For both study arms, we will collect archival cost information for two years before the intervention (pre-randomization) and two years from the start of the intervention implementation (post-randomization). Thus, the additional costs of NIATx will be the difference between the pre-post program change in costs and the pre-post control program change in costs. Change in costs will be calculated as:

Pre-post change in costs = (Total Costs)_post_ – (Total Costs)_pre_ - (Total Revenue)_post_ –(Total Revenue)_pre_.

The impact of improved integrated services could lead to additional (reflected as decreased costs) or lost (reflected as additional costs) revenues. By dividing each component by the number of admissions in that period, the net cost per admission can be derived. The economic analysis will identify important sustainability implications and could influence future stakeholder implementation decisions [76].

## Dissemination policy

Irrespective of the magnitude or direction of NIATx strategy effect, we will disseminate study findings. Dissemination efforts will include presentations at professional scientific conferences and publication in peer-reviewed journals with the highest impact factor possible. Additionally, we will seek to ensure the project’s publications are open access (i.e., available online to readers without financial, legal, or technical barriers beyond those inseparable from gaining access to the internet).

## Discussion

The use of NIATx implementation strategies spread beyond efforts to improve access and retention to address organizational change efforts to reduce psychiatric re-admissions [77], support implementation of evidence based-practices such as Seeking Safety [78] or trauma informed care [79], and improve no-show rates [80]. In addition, NIATx or NIATx-like implementation strategies have been utilized to improve process of care in drug courts [81] and explore the impact of feedback reports in residential treatment organizations [82]. Similar to the original NIATx studies, the efforts represent an application of NIATx implementation strategies in a single setting (e.g., mental health), targeting a specific outcome (e.g., no-shows).

Recent studies have explored how NIATx or NIATx-like implementation strategies support organizational change efforts for more complex patients in specialized environments. Examples include changes targeting HIV treatment in correctional settings [83–86] and the implementation of evidence-based prevention practices for older adults in community health settings [87, 88]. Other studies have integrated NIATx implementation strategies with external policy and regulatory levers to improve access to medications for alcohol and opioid use disorders [59, 89, 90].

The proposed study represents a substantial advance in addressing a gap in the existing NIATx implementation research. This research represents the first true test of NIATx for implementing complex, not simple, treatment services in substance use programs and evaluates how the use of NIATx implementation strategies improves services for individuals with co-occurring disorders. The proposed study accomplishes this objective by unifying and operationalizing objective measures across three types of implementation frameworks (determinant, evaluative and process) to address a longstanding gap in the quality of care for persons with co-occurring disorders. Specifically, it explores the relationship between use of NIATx strategies and implementation and patient level outcomes. It is the first study of NIATx implementation strategies to include a specific aim to precisely document the fidelity with which NIATx is delivered. The modified NIATx Stages of Implementation Completion will explore the extent of activity completion of the purportedly essential components of NIATx using the Stages of Implementation Completion approach. Neither the NIATx research platform nor community users have thus far experienced this rigorous a level of scientific inquiry. Findings from this research can be immediately applied to improve clinical services, advance implementation research, as well as expand and guide research with other systems and settings.

## Trial status

The trial has been determined to not involve human subjects research. As of 18 January 2018, 53 addiction treatment agencies volunteered or were recruited to be in the study, and 49 were randomized.

## Additional file

Additional file 1: NIATx Protocol Data Dictionary. (PDF 62 kb)

## Abbreviations

ASI: Addiction Severity Index
BHDS: Behavioral Health Data System
CFIR: Consolidated Framework for Implementation Research
DBHR: Division of Behavioral Health and Recovery
DDCAT: Dual Diagnosis Capability in Addiction Treatment
GSS: Gain Short Screener
MCO: Managed Care Organizations
MH: Mental Health
MH-CIS: Mental Health Consumer Information System
NIATx: Network for the Improvement of Addiction Treatment
NIDA: National Institute of Drug Abuse
RCT: Randomized Control Trial
SIC: Stages of Implementation Completion
SPIRIT: Standard Protocol Items: Recommendation for Intervention Trials
SUD: Substance Use Disorders
TARGET: Treatment and Assessment Reports Generation Tool
