# Supplementary material for: Using NIATx strategies to implement integrated services in routine care: a study protocol
Source: BMC Health Serv Res. 2018 Jun 8;18:431. doi: 10.1186/s12913-018-3241-4 (PMC5994046; doi:10.1186/s12913-018-3241-4)
Supplement: Supplementary file 1 — NIATx Protocol Data Dictionary. (PDF 62 kb) [file 12913_2018_3241_MOESM1_ESM.pdf]

Member Name	Notes
Demographic information from BHDS for baseline and 1yr f/u	
FINAL_BHDS_DEMOGS	
FINAL_BHDS_DEMOGS	M or F
FINAL_BHDS_DEMOGS	Y or N
FINAL_BHDS_DEMOGS	text descriptor or race
FINAL_BHDS_DEMOGS	
MAT rx from BHDS for baseline and 1yr f/u. Link to FINAL_BHDS_NIATX_PGM_ADMITS on uniqueid where mat_rx_day is between admission and the 90-days following admission.	
FINAL_BHDS_MAT_NIATX	MAT drug name
FINAL_BHDS_MAT_NIATX	date of MAT rx
FINAL_BHDS_MAT_NIATX	
CD and MH Encounters from BHDS for baseline and 1yr f/u. Link to FINAL_BHDS_NIATX_PGM_ADMITS on uniqueid and provider where servdate is between admission and the 90-days following admission.	
FINAL_BHDS_NIATX_ENCOUNTERS	CD or MH
FINAL_BHDS_NIATX_ENCOUNTERS	(N)iatx or (W)aitlist
FINAL_BHDS_NIATX_ENCOUNTERS	Text descriptor of SERI service
FINAL_BHDS_NIATX_ENCOUNTERS	Text descriptor of NIATX service roll-up
FINAL_BHDS_NIATX_ENCOUNTERS	Provider Name
FINAL_BHDS_NIATX_ENCOUNTERS	service date
FINAL_BHDS_NIATX_ENCOUNTERS	Baseline or 1 yr follow-up
FINAL_BHDS_NIATX_ENCOUNTERS	
Psycotropic rx from BHDS for baseline and 1yr f/u. Link to FINAL_BHDS_NIATX_PGM_ADMITS on uniqueid where psycotropic_rx_day is between admission and the 90-days following admission.	
FINAL_BHDS_NIATX_MH_RX	date of psycotropic rx
FINAL_BHDS_NIATX_MH_RX	
BHDS admissions (and constructed episodes) for baseline and 1yr f/u.	
FINAL_BHDS_NIATX_PGM_ADMITS	Admission Date
FINAL_BHDS_NIATX_PGM_ADMITS	Service Episode (ServiceEpi), Program Enrollment(PgmEnroll), or Calculated Episode (CalcEpi)
FINAL_BHDS_NIATX_PGM_ADMITS	Discharge Date

FINAL_BHDS_NIATX_PGM_ADMITS	(N)iatx or (W)aitlist
FINAL_BHDS_NIATX_PGM_ADMITS	Provider Name
FINAL_BHDS_NIATX_PGM_ADMITS	Baseline or 1 yr follow-up
FINAL_BHDS_NIATX_PGM_ADMITS	
Pre-Baseline TARGET admits	
FINAL_TARGET_ADMITS	
FINAL_TARGET_ADMITS	
FINAL_TARGET_ADMITS	see TARGET data dictionary (1)
FINAL_TARGET_ADMITS	(N)iatx or (W)aitlist
FINAL_TARGET_ADMITS	see TARGET data dictionary (1)
FINAL_TARGET_ADMITS	Provider Name
FINAL_TARGET_ADMITS	
Pre-Baseline TARGET ASI variables. Link to FINAL_TARGET_ADMITS by uniqueid and admission_datetime	
FINAL_TARGET_ASI	see TARGET data dictionary (1)
FINAL_TARGET_ASI	see TARGET data dictionary (1)
FINAL_TARGET_ASI	see TARGET data dictionary (1)
FINAL_TARGET_ASI	see TARGET data dictionary (1)
FINAL_TARGET_ASI	see TARGET data dictionary (1)
FINAL_TARGET_ASI	see TARGET data dictionary (1)
FINAL_TARGET_ASI	see TARGET data dictionary (1)
FINAL_TARGET_ASI	see TARGET data dictionary (1)
FINAL_TARGET_ASI	see TARGET data dictionary (1)
FINAL_TARGET_ASI	see TARGET data dictionary (1)
FINAL_TARGET_ASI	see TARGET data dictionary (1)
FINAL_TARGET_ASI	see TARGET data dictionary (1)
FINAL_TARGET_ASI	(N)iatx or (W)aitlist
FINAL_TARGET_ASI	see TARGET data dictionary (1)
FINAL_TARGET_ASI	see TARGET data dictionary (1)
FINAL_TARGET_ASI	Provider Name
FINAL_TARGET_ASI	see TARGET data dictionary (1)
FINAL_TARGET_ASI	see TARGET data dictionary (1)
FINAL_TARGET_ASI	see TARGET data dictionary (1)
FINAL_TARGET_ASI	see TARGET data dictionary (1)

FINAL_TARGET_ASI	see TARGET data dictionary (1)
FINAL_TARGET_ASI	see TARGET data dictionary (1)
FINAL_TARGET_ASI	see TARGET data dictionary (1)
FINAL_TARGET_ASI	see TARGET data dictionary (1)
FINAL_TARGET_ASI	
Pre-Baseline TARGET encounters. Link to FINAL_TARGET_ADMITS by uniqueid and provider where the servdate is between admission and the 90-days following admission.	
FINAL_TARGET_ENCOUNTERS	CD or MH
FINAL_TARGET_ENCOUNTERS	(N)iatx or (W)aitlist
FINAL_TARGET_ENCOUNTERS	Text descriptor of SERI service
FINAL_TARGET_ENCOUNTERS	Provider Name
FINAL_TARGET_ENCOUNTERS	service date
FINAL_TARGET_ENCOUNTERS	Text descriptor of NIATX service roll-up
FINAL_TARGET_ENCOUNTERS	
Pre-Baseline TARGET gain scores. Link to FINAL_TARGET_ADMITS by uniqueid and admission_datetime	
FINAL_TARGET_GAIN	
FINAL_TARGET_GAIN	see TARGET data dictionary (1)
FINAL_TARGET_GAIN	date GAIN was collected
FINAL_TARGET_GAIN	(N)iatx or (W)aitlist
FINAL_TARGET_GAIN	see TARGET data dictionary (1)
FINAL_TARGET_GAIN	Provider Name
FINAL_TARGET_GAIN	see TARGET data dictionary (1)
FINAL_TARGET_GAIN	
Pre-Baseline TARGET demographics. Link to FINAL_TARGET_ADMITS by uniqueid.	
FINAL_TARGET_IDS	
FINAL_TARGET_IDS	M or F
FINAL_TARGET_IDS	Y or N
FINAL_TARGET_IDS	text descriptor or race
FINAL_TARGET_IDS	
Pre-Baseline TARGET MAT. Link to FINAL_TARGET_ADMITS by uniqueid where the mat_rx_day is between admission and the 90-days following admission.	
FINAL_TARGET_MAT	MAT drug name

FINAL_TARGET_MAT	date of MAT rx
FINAL_TARGET_MAT	
Pre-Baseline TARGET psycotropic rx. Link to FINAL_TARGET_ADMITS by uniqueid where the psycotropic_rx_day is between admission and the 90-days following admission.	
FINAL_TARGET_NIATX_MH_RX	date of psycotropic rx
FINAL_TARGET_NIATX_MH_RX	
(1) https://www.dshs.wa.gov/sites/default/files/BHSIA/dbh/documents/__Data%20Dictionary_Massive.pdf	
